# No Association Between HLH-associated Gene Variants and Life-Threatening COVID-19

**DOI:** 10.1007/s10875-025-01870-6

**Published:** 2025-03-11

**Authors:** Laura E. Covill, Aurélie Cobat, COVID Human Genetic Effort, Qian Zhang, Yenan T. Bryceson

**Affiliations:** 1 Center for Hematology and Regenerative Medicine, Department of Medicine, Karolinska Institute, Karolinska University Hospital Huddinge, Stockholm, Sweden; 2 St Giles Laboratory of Human Genetics of Infectious Diseases, Rockefeller Branch, Rockefeller University, New York, NY USA; 3 Laboratory of Human Genetics of Infectious Diseases, Necker Branch, INSERM U1163, Necker Hospital for Sick Children, Paris, France; 4 University Paris Cité, Imagine Institute, Paris, France; 5 Clinical Immunology and Transfusion Medicine, Karolinska University Hospital, Stockholm, Sweden; 6 Brogelmann Research Laboratory, Department of Clinical Science, University of Bergen, Bergen, Norway

To the editor,

COVID-19 mortality is often accompanied by excessive proinflammatory cytokine release, termed “cytokine storm”. Hemophagocytic lymphohistiocytosis (HLH) is a life-threatening hyperinflammatory syndrome that shares some clinical and laboratory features with COVID-19 cytokine storm. Providing notable parallels to the treatment of HLH, immunosuppressive glucocorticoid therapies are efficacious in severe COVID-19 patients [1].

The majority of familial HLH cases present in infancy and are caused by autosomal recessive loss-of-function (LoF) variants in genes required for lymphocyte cytotoxicity (*PRF1*, *UNC13D*, *STX11*, *STXBP2*, *RAB27A*, *LYST*, *AP3B1*, *RHOG;* Supplementary Table 1). In addition, HLH is a common feature of children with certain actinopathies or inflammasome activation syndromes (*NCKAP1L*, *CDC42*, *NLRC4*; the two latter associated with dominant gain-of-function variants) as well as autosomal recessive LoF variants in genes required for type I IFN signaling (*IFNAR1*, *IFNAR2*, *STAT1*, *STAT2*). In contrast to primary HLH, secondary forms of HLH lacking strong genetic components occur more often in adulthood. Viral infections are frequent triggers of primary as well as secondary HLH. Heterozygous genetic variants in genes causative of familial HLH may also contribute to secondary HLH or cancer predisposition. The pathogenicity of these genes may therefore extend beyond early-onset disease caused by biallelic LoF variants to haploinsufficiency increasing susceptibility to severe hyperinflammatory disease upon viral infection. It has been speculated that HLH and severe COVID-19 might have an overlapping genetic pathogenesis. A previous single-center investigation of 233 COVID-19 patients reported enrichment of rare *UNC13D* and *AP3B1* variants in 42 critically ill patients, with variants correlating to cytokinemia [2]. Limitations of this study included a small sample size, a high average age of patients giving a possible confounding variable, and underrepresentation in population databases of the ethnic group included in the study, causing difficulty in filtering genuinely rare variants in these groups. A single severe COVID-19 patient with a heterozygous *STXBP2* missense variant that suppresses cytotoxic lymphocyte exocytosis in a dominant negative manner has also been reported [3]. Here, we analyzed a larger cohort and considered ethnicity as a covariate in our model to evaluate the potential contribution of variants in genes strongly associated with HLH to critical COVID-19 susceptibility.

The COVID Human Genetic Effort (CHGE) has assembled a large cohort of SARS-CoV-2 serum-positive individuals with varying disease severity analyzed by exome or genome sequencing. We investigated whether exonic coding and splicing variants with rare minor allele frequency (MAF) in gnomAD v2 in 15 genes associated with HLH were enriched in exomes from 3269 critically ill patients relative to 1373 asymptomatic or mild COVID-19 cases (referred to as ‘controls’) who were roughly age matched (Fig. 1A, B), using previously reported gene burden analysis [4]. As the controls were biased towards European ethnicity (Fig. 1C), odds ratios (ORs) were adjusted by the first five ancestry principal components (PCs) for each group. Overall, no enrichment of rare variants was observed in critical cases when adjusted for ancestry (Fig. 1D), suggesting that being a heterozygous carrier of such rare variants in these genes does not significantly predispose to life-threatening COVID-19. Rather, in rare cases, biallelic deficiency of *IFNAR1*, *IFNAR2*, and *STAT1* have been identified in patients with life-threatening COVID-19, but thus far there are no reports of life-threatening COVID-19 in patients with biallelic LoF variants in genes required for lymphocyte cytotoxicity.

**Fig. 1:**
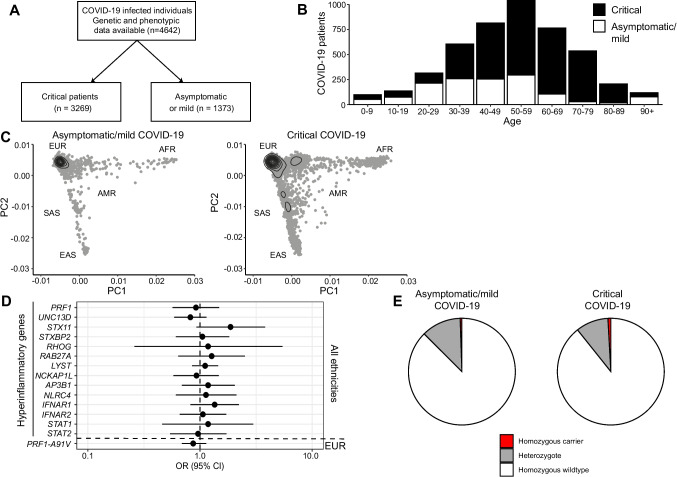
**A** Patients from the COVID Human Genetic Cohort who had genotype information available and were either critical or asymptomatic or mild in presentation were included in the study. **B** Age distribution of individuals with asymptomatic or mild or critical COVID-19. **C** Ancestry principal components analyses of COVID-19 subjects and controls. Contour plots of the first two PCs are shown for (L-to-R) the 1kGP cohort; asymptomatic or mild COVID-19 controls included in the study; critical cases of COVID-19 included in the study. **D** Forrest plot of odds ratios for enrichment of variants with MAF < 0.001 in cases compared to controls for each of the 15 genes considered in the study, as reported previously [4]. Additionally, an odds ratio was calculated in the European study participants only for enrichment of the *PRF1* c.272C > T (p.A91V) risk variant, included in the same Forrest plot.** E** Pie charts show the proportions of control (left) and critically infected (right) individuals of European descent who carried the *PRF1* p.A91V variant in heterozygous or homozygous form

We also evaluated a common hypomorphic risk variant for HLH, *PRF1* c.272C > T (p.Ala91Val), examining critical COVID-19 patients versus controls. This variant, conferring approximately 50% of normal perforin function, is most common in non-Finnish Europeans with MAF of 4.6% in gnomAD v2. Thus, only 2331 CHGE cases falling within principal component boundaries for European individuals taken from the 1000 Genomes Project (1kGP) were included in these analyses. Of these, 959 were controls and 1372 were severe or critical cases. One hundred and twenty of 959 (including five homozygotes; MAF = 6.5%) controls and 135 of 1372 (including twelve homozygotes; MAF = 5.8%) critical cases were carriers of *PRF1* p.Ala91Val (OR = 0.87, p = 0.29; Fig. 1D). Given that age is a risk factor for critical COVID-19, we also examined relative age in this cohort. The average age of the critical carriers was 62.4 years, compared to 47.7 years amongst controls (p < 0.01, Welch T-test), which was representative of the average ages amongst the total critical cases and controls of European ancestry. Together, these analyses do not indicate that this hypomorphic variant in *PRF1* confers a major risk of life-threatening COVID-19.

Since HLH-associated genetic diseases are recessive, we attempted to identify cases from the CHGE cohort where biallelic rare variants could be present. Suspected cases where biallelic variants with MAF < 0.01 were evaluated on a case-by-case basis to establish the likelihood of compound heterozygosity. Where variants had been observed on the same haplotype in gnomAD or were predicted to appear on the same haplotype using the variant co-occurrence tool with a probability greater than 50%, we assumed monoallelic carriership. If one or both variants were not present in gnomAD, compound heterozygosity was assumed. Of 203 individuals in the CHGE cohort carrying two or more variants in an HLH-associated gene, 87 were estimated to be monoallelic with both variants occurring on the same haplotype. Of the remaining 116 cases, 86 (2.63%) of the critical COVID-19 patients were predicted to carry biallelic variants with gnomAD MAF < 0.01 in one of 15 genes associated with HLH and 30 (2.18%) controls were predicted to carry biallelic variants. Notably, the proportion of non-European biallelic carriers does not represent a departure from the overall cohort for either the critical group (42.0% European ancestry overall, and 39.5% of the biallelic carriers), or the control carriers (69.9% of the controls were of European ancestry, compared to 70.0% of those predicted to carry two rare variants in an HLH-associated gene). Thus, a better ethnic representation among the controls would not be expected to increase the proportion carrying biallelic HLH-associated variants. Nonetheless, poor long-term clinical outcomes associated with the potential of functional biallelic LoF variants in HLH-associated genes, including recurrent or prolonged hyperinflammatory episodes and possible susceptibility to further viral infections, would suggest that functional testing could be warranted in critically ill patients with biallelic rare variants. Degranulation assays or testing of the type I IFN signaling pathway can be advantageous to establish if they have relevant inborn errors of immunity.

In summary, we did not find that being a carrier of rare heterozygous variants, including the hypomorphic *PRF1* p.Ala91Val variant, were associated with an increased risk of life-threatening COVID-19 in the general population. Although we cannot exclude that rare biallelic variants in HLH-associated genes may predispose to severe COVID-19, our findings as well as a paucity of reports of COVID-19 associated with familial HLH patients in the medical literature suggests that genetic deficiencies in lymphocyte cytotoxicity are not major factors in predisposing to critical COVID-19 upon SARS-CoV-2 infection. We also infer from this finding that heterozygous relatives of familial HLH patients are not at increased risk of critical COVID-19. In fact, a study examining coronavirus infection causing hepatitis in mice found that *Prf1* knock-out mice displayed slightly prolonged survival compared to wild-type mice [5]. It is therefore likely that lymphocyte cytotoxicity does not represent a crucial factor for effective control of SARS-CoV2 infection, which rather relies on plasmacytoid dendritic cells and the type I IFN-mediated immunity.

## Supplementary Information

Below is the link to the electronic supplementary material.

Supplementary file1 (DOCX 272 KB)

## Data Availability

No datasets were generated or analysed during the current study.
