# Supplementary material for: No Association Between HLH-associated Gene Variants and Life-Threatening COVID-19
Source: J Clin Immunol. 2025 Mar 11;45(1):80. doi: 10.1007/s10875-025-01870-6 (PMC11897064; doi:10.1007/s10875-025-01870-6)
Supplement: Supplementary file 1 — Supplementary file1 (DOCX 272 KB) [file 10875_2025_1870_MOESM1_ESM.docx]

**Supplementary Materials and Methods**

*Cohort*

The CHGE is a consortium of international labs and clinicians who have collected and sequenced a large cohort for the purpose of defining genetic and immunological causes of critical COVID-19 (1). The full cohort, comprising individuals with heterogenous clinical phenotypes in response to SARS-CoV-2 infection, numbers around 10,000. Patients with autoantibodies to Type 1 interferon, or where phenotypic evaluation was not available, were excluded from analysis.

*Sequencing*

Sequencing and preprocessing of the CHGE cohort was described in Zhang et al., 2020 (1).

*Ancestry analysis*

Ancestry PCs were calculated using plink2 and mapped to the 1000 Genomes Project for quality control (2). The first 5 PCs were used as covariates in OR calculation.

*Rare variant extraction*

Variants with MAF less than 0.01 were extracted from whole exome and whole genome sequencing data in *PRF1*, *UNC13D*, *STX11*, *STXBP2*, *RAB27A*, *LYST*, *AP3B1*, *RHOG*, *NCKAP1L*, *CDC42*, *NLRC4*, *IFNAR1*, *IFNAR2*, *STAT1*, and *STAT2*, which were used to identify individuals with possible biallelic carriership. For OR calculations, further filtering was performed to identify variants with MAF less than 0.001, as previously described (3).

*Statistics*

Statistical analyses were performed using R (v4.1.1). For odds ratio calculations, the logistf package was used to perform penalized likelihood ratio tests.

**Supplementary Table 1. Hyperinflammatory syndromes associated with genes included in the study.**

| **Hyperinflammatory syndrome-**  **associated gene** | **Reference** |
| --- | --- |
| *PRF1* | (4) |
| *UNC13D* | (5) |
| *STX11* | (6) |
| *STXBP2* | (7) |
| *RHOG* | (8) |
| *AP3B1* | (9) |
| *RAB27A* | (10) |
| *LYST* | (11) |
| *NLRC4* | (12) |
| *NCKAP1L* | (13) |
| *CDC42* | (14) |
| *IFNAR1* | (15) |
| *IFNAR2* | (16) |
| *STAT1* | (17) |
| *STAT2* | (18) |

**Supplementary Methods References**

1. Zhang Q, Liu Z, Moncada-Velez M, Chen J, Ogishi M, Bigio B, et al. Inborn errors of type I IFN immunity in patients with life-threatening COVID-19. Science (1979). 2020 Oct 23;370(6515).

2. Auton A, Abecasis GR, Altshuler DM, Durbin RM, Bentley DR, Chakravarti A, et al. A global reference for human genetic variation. Vol. 526, Nature. Nature Publishing Group; 2015. p. 68–74.

3. Matuozzo D, Talouarn E, Marchal A, Zhang P, Manry J, Seeleuthner Y, et al. Rare predicted loss-of-function variants of type I IFN immunity genes are associated with life-threatening COVID-19. Genome Med. 2023 Dec 1;15(1).

4. Stepp SE, Dufourcq-Lagelouse R, Deist F Le, Bhawan S, Certain S, Mathew PA, et al. Perforin Gene Defects in Familial Hemophagocytic Lymphohistiocytosis. Science (1979). 1999 Dec 3;286(5446):1957–9.

5. Feldmann J, Callebaut I, Raposo G, Certain S, Bacq D, Dumont C, et al. Munc13-4 Is Essential for Cytolytic Granules Fusion and Is Mutated in a Form of Familial Hemophagocytic Lymphohistiocytosis (FHL3) [Internet]. Vol. 115, Cell. 2003. Available from: www.ncbi.nlm.nih.gov/mapview

6. zur Stadt U, Schmidt S, Kasper B, Beutel K, Diler AS, Henter JI, et al. Linkage of familial hemophagocytic lymphohistiocytosis (FHL) type-4 to chromosome 6q24 and identification of mutations in syntaxin 11. Hum Mol Genet. 2005 Mar 15;14(6):827–34.

7. zur Stadt U, Rohr J, Seifert W, Koch F, Grieve S, Pagel J, et al. Familial Hemophagocytic Lymphohistiocytosis Type 5 (FHL-5) Is Caused by Mutations in Munc18-2 and Impaired Binding to Syntaxin 11. Am J Hum Genet. 2009 Oct 9;85(4):482–92.

8. Kalinichenko A, Perinetti Casoni G, Dupré L, Trotta L, Huemer J, Galgano D, et al. RhoG deficiency abrogates cytotoxicity of human lymphocytes and causes hemophagocytic lymphohistiocytosis. Blood. 2021 Apr 15;137(15).

9. Enders A, Zieger B, Schwarz K, Yoshimi A, Speckmann C, Knoepfle EM, et al. Lethal hemophagocytic lymphohistiocytosis in Hermansky-Pudlak syndrome type II. Blood. 2006 Jul 1;108(1):81–7.

10. Ménasché G, Pastural E, Feldmann J, Certain S, Ersoy F, Dupuis S, et al. Mutations in RAB27A cause Griscelli syndrome associated with haemophagocytic syndrome. Nat Genet [Internet]. 2000;25:173. Available from: http://genetics.nature.com

11. Nagle DL, Karim MA, Woolf EA, Holmgren L, Bork3 P, Misumi1 DJ, et al. Identification and mutation analysis of the complete gene for Chediak-Higashi syndrome. Nat Genet [Internet]. 1996;14:307–11. Available from: http://www.nature.com/naturegenetics

12. Canna SW, De Jesus AA, Gouni S, Brooks SR, Marrero B, Liu Y, et al. An activating NLRC4 inflammasome mutation causes autoinflammation with recurrent macrophage activation syndrome. Nat Genet. 2014 Sep 26;46(10):1140–6.

13. Castro CN, Rosenzwajg M, Carapito R, Shahrooei M, Konantz M, Khan A, et al. NCKAP1L defects lead to a novel syndrome combining immunodeficiency, lymphoproliferation, and hyperinflammation. Journal of Experimental Medicine. 2020 Aug 6;217(12).

14. Lam MT, Coppola S, Krumbach OHF, Prencipe G, Insalaco A, Cifaldi C, et al. A novel disorder involving dyshematopoiesis, inflammation, and HLH due to aberrant CDC42 function. Journal of Experimental Medicine. 2019 Dec 1;216(12):2778–99.

15. Gothe F, Hatton CF, Truong L, Klimova Z, Kanderova V, Fejtkova M, et al. A Novel Case of Homozygous Interferon Alpha/Beta Receptor Alpha Chain (IFNAR1) Deficiency With Hemophagocytic Lymphohistiocytosis. Clinical Infectious Diseases. 2022 Jan 1;74(1):136–9.

16. Passarelli C, Civino A, Rossi MN, Cifaldi L, Lanari V, Moneta GM, et al. IFNAR2 Deficiency Causing Dysregulation of NK Cell Functions and Presenting With Hemophagocytic Lymphohistiocytosis. Front Genet. 2020 Sep 18;11.

17. Boehmer DFR, Koehler LM, Magg T, Metzger P, Rohlfs M, Ahlfeld J, et al. A Novel Complete Autosomal-Recessive STAT1 LOF Variant Causes Immunodeficiency with Hemophagocytic Lymphohistiocytosis–Like Hyperinflammation. Journal of Allergy and Clinical Immunology: In Practice. 2020 Oct 1;8(9):3102–11.

18. Gothe F, Stremenova Spegarova J, Hatton CF, Griffin H, Sargent T, Cowley SA, et al. Aberrant inflammatory responses to type I interferon in STAT2 or IRF9 deficiency. Journal of Allergy and Clinical Immunology. 2022 Oct 1;150(4):955-964.e16.

**Appendix 1**

**Members of the COVID Human Genetic Effort**

Laurent Abel^1^, Alessandro Aiuti^2^, Saleh Al-Muhsen^3^, Fahd Al-Mulla^4^, Mark S. Anderson^5^, Evangelos Andreakos^6^, Andrés A. Arias^7^, Hagit Baris Feldman^8^, Alexandre Belot^9^, Catherine M. Biggs^10^, Dusan Bogunovic^11^, Alexandre Bolze^12^, Anastasiia Bondarenko^13^, Ahmed A. Bousfiha^14^, Petter Brodin^15^, Yenan Bryceson^16^, Carlos D. Bustamante^17^, Manish J. Butte^18^, Giorgio Casari^19^, John Christodoulou^20^, Roger Colobran^21^, Antonio Condino-Neto^22^, Stefan N. Constantinescu^23^, Megan A. Cooper^24^, Clifton L. Dalgard^25^, Murkesh Desai^26^, Beth A. Drolet^27^, Jamila El Baghdadi^28^, Sara Espinosa-Padilla^29^, Jacques Fellay^30^, Carlos Flores^31^, José Luis Franco^32^, Antoine Froidure^33^, Peter K. Gregersen^34^, Bodo Grimbacher^35^, Filomeen Haerynck^36^, David Hagin^37^, Rabih Halwani^38^, Lennart Hammarström^39^, James R. Heath^40^, Sarah E. Henrickson^41^, Elena W.Y. Hsieh^42^, Eystein Husebye^43^, Kohsuke Imai^44^, Yuval Itan^45^, Erich D. Jarvis^46^, Timokratis Karamitros^47^, Kai Kisand^48^, Cheng-Lung Ku^49^, Yu-Lung Lau^50^, Yun Ling^51^, Carrie L. Lucas^52^, Davood Mansouri^53^, László Maródi^54^, Isabelle Meyts^55^, Joshua D. Milner^56^, Kristina Mironska^57^, Trine H. Mogensen^58^, Tomohiro Morio^59^, Lisa F.P. Ng^60^, Luigi D. Notarangelo^61^, Antonio Novelli^62^, Giuseppe Novelli^63^, Cliona O'Farrelly^64^, Satoshi Okada^65^, Keisuke Okamoto^66^, Tayfun Ozcelik^67^, Qiang Pan-Hammarström^38^, Jean W. Pape^68^, Rebeca Perez de Diego^69^, David S. Perlin^70^, Graziano Pesole^71^, Anna M. Planas^72^, Carolina Prando^73^, Aurora Pujol^74^, Lluis Quintana-Murci^75^, Sathishkumar Ramaswamy^76^, Laurent Renia^60^, Igor Resnick^77^, Carlos Rodríguez-Gallego^78^, Vanessa Sancho-Shimizu^79^, Anna Sediva^80^, Mikko R.J. Seppänen^81^, Mohammed Shahrooei^82^, Anna Shcherbina^83^, Ondrej Slaby^84^, Andrew L. Snow^85^, Pere Soler-Palacín^86^, András N. Spaan^87^, Ivan Tancevski^88^, Stuart G. Tangye^89^, Ahmad Abou Tayoun^76^, Şehime Gülsün Temel^90^, Stuart E. Turvey^91^, K M Furkan Uddin^92^, Mohammed J. Uddin^93^, Diederik van de Beek^94^, Donald C. Vinh^95^, Horst von Bernuth^96^, Joost Wauters^97^, Mayana Zatz^98^, Pawel Zawadzki^99^, Helen C. Su^61^, Jean-Laurent Casanova^100^

1. Laboratory of Human Genetics of Infectious Diseases, Necker Branch, INSERM U1163, Necker Hospital for Sick Children, Paris, France; University of Paris, Imagine Institute, Paris, France.

2. San Raffaele Telethon Institute for Gene Therapy, IRCCS Ospedale San Raffaele, and Vita Salute San Raffaele University, Milan, Italy.

3. Immunology Research Lab, Department of Pediatrics, College of Medicine, King Saud University, Riyadh, Saudi Arabia.

4. Dasman Diabetes Institute, Department of Genetics and Bioinformatics, Dasman, Kuwait.

5. Diabetes Center, University of California San Francisco, San Francisco, CA, USA.

6. Laboratory of Immunobiology, Center for Clinical, Experimental Surgery and Translational Research, Biomedical Research Foundation of the Academy of Athens, Athens, Greece.

7. St. Giles Laboratory of Human Genetics of Infectious Diseases, Rockefeller Branch, The Rockefeller University, New York, NY, USA; Primary Immunodeficiencies Group, Department of Microbiology and Parasitology, School of Medicine, University of Antioquia, Medellín, Colombia; School of Microbiology, University of Antioquia UdeA, Medellín, Colombia.

8. The Genetics Institute, Tel Aviv Sourasky Medical Center and Sackler Faculty of Medicine, Tel Aviv University, Tel Aviv, Israel.

9. Pediatric Nephrology, Rheumatology, Dermatology, HFME, Hospices Civils de Lyon, National Referee Centre RAISE, and INSERM U1111, Université de Lyon, Lyon, France.

10. Department of Pediatrics, BC Children's and St. Paul's Hospitals, University of British Columbia, Vancouver, BC, Canada.

11. Icahn School of Medicine at Mount Sinai, New York, NY, USA.

12. Helix, San Mateo, CA, USA.

13. Shupyk National Medical Academy for Postgraduate Education, Kiev, Ukraine.

14. Clinical Immunology Unit, Department of Pediatric Infectious Disease, CHU Ibn Rushd and LICIA, Laboratoire d'Immunologie Clinique, Inflammation et Allergie, Faculty of Medicine and Pharmacy, Hassan II University, Casablanca, Morocco.

15. SciLifeLab, Department Of Women’s and Children’s Health, Karolinska Institutet, Stockholm, Sweden.

16. Department of Medicine, Center for Hematology and Regenerative Medicine, Karolinska Institutet, Stockholm, Sweden.

17. Stanford University, Stanford, CA, USA.

18. Division of Immunology, Allergy, and Rheumatology, Department of Pediatrics and the Department of Microbiology, Immunology, and Molecular Genetics, University of California, Los Angeles, CA, USA.

19. Clinical Genomics, IRCCS San Raffaele Scientific Institute and Vita-Salute San Raffaele University, Milan, Italy.

20. Murdoch Children's Research Institute and Department of Paediatrics, University of Melbourne, Melbourne, VIC, Australia.

21. Immunology Division, Vall d’Hebron Barcelona Hospital Campus, Barcelona, Catalonia, Spain.

22. Department of Immunology, Institute of Biomedical Sciences, University of São Paulo, São Paulo, Brazil.

23. de Duve Institute and Ludwig Cancer Research, Brussels, Belgium.

24. Washington University School of Medicine, St. Louis, MO, USA.

25. Department of Anatomy, Physiology & Genetics, Uniformed Services University of the Health Sciences, Bethesda, MD, USA.

26. Bai Jerbai Wadia Hospital for Children, Mumbai, India.

27. School of Medicine and Public Health, University of Wisconsin, Madison, WI, USA.

28. Genetics Unit, Military Hospital Mohamed V, Rabat, Morocco.

29. Instituto Nacional de Pediatria (National Institute of Pediatrics), Mexico City, Mexico.

30. School of Life Sciences, Ecole Polytechnique Fédérale de Lausanne, Lausanne, Switzerland; Precision Medicine Unit, Lausanne University Hospital and University of Lausanne, Lausanne, Switzerland.

31. Research Unit, Hospital Universitario Nuestra Señora de Candelaria, Santa Cruz de Tenerife; CIBER de Enfermedades Respiratorias, Instituto de Salud Carlos III, Madrid; Genomics Division, Instituto Tecnológico y de Energías Renovables (ITER), Santa Cruz de Tenerife, Spain.

32. Group of Primary Immunodeficiencies, University of Antioquia UDEA, Medellin, Colombia.

33. Pulmonology Department, Cliniques Universitaires Saint-Luc ; Institut de Recherche Expérimentale et Clinique (IREC), Université Catholique de Louvain, Brussels, Belgium.

34. Feinstein Institute for Medical Research, Northwell Health USA, Manhasset, NY, USA.

35. Center for Chronic Immunodeficiency & Institute for Immunodeficiency, Medical Center, Faculty of Medicine, University of Freiburg, Freiburg, Germany.

36. Department of Paediatric Immunology and Pulmonology, Centre for Primary Immunodeficiency Ghent (CPIG), PID Research Laboratory, Jeffrey Modell Diagnosis and Research Centre, Ghent University Hospital, Ghent, Belgium.

37. The Genetics Institute Tel Aviv Sourasky Medical Center, Tel Aviv, Israel.

38. Sharjah Institute of Medical Research, College of Medicine, University of Sharjah, Sharjah, United Arab Emirates.

39. Department of Biosciences and Nutrition, Karolinska Institutet, Stockholm, Sweden.

40. Institute for Systems Biology, Seattle, WA, USA.

41. Department of Pediatrics, Division of Allergy Immunology, Children’s Hospital of Philadelphia, Philadelphia, PA, USA; Department of Microbiology, Perelman School of Medicine, University of Pennsylvania, Philadelphia, PA, USA.

42. Departments of Pediatrics, Immunology and Microbiology, University of Colorado, School of Medicine, Aurora, CO, USA.

43. Department of Medicine, Haukeland University Hospital, Bergen, Norway.

44. Department of Community Pediatrics, Perinatal and Maternal Medicine, Tokyo Medical and Dental University (TMDU), Tokyo, Japan.

45. Institute for Personalized Medicine, Icahn School of Medicine at Mount Sinai, New York, NY, USA; Department of Genetics and Genomic Sciences, Icahn School of Medicine at Mount Sinai, New York, NY, USA.

46. Laboratory of Neurogenetics of Language and Howard Hughes Medical Institute, The Rockefeller University, New York, NY, USA.

47. Bioinformatics and Applied Genomics Unit, Hellenic Pasteur Institute, Athens, Greece.

48. Molecular Pathology, Department of Biomedicine, Institute of Biomedicine and Translational Medicine, University of Tartu, Tartu Estonia.

49. Chang Gung University, Taoyuan County, Taiwan.

50. Department of Paediatrics & Adolescent Medicine, The University of Hong Kong, Hong Kong, China.

51. Shanghai Public Health Clinical Center, Fudan University, Shanghai, China.

52. Department of Immunobiology, Yale University School of Medicine, New Haven, CT, USA.

53. Department of Clinical Immunology and Infectious Diseases, National Research Institute of Tuberculosis and Lung Diseases, The Clinical Tuberculosis and Epidemiology Research Center, National Research Institute of Tuberculosis and Lung Diseases (NRITLD), Masih Daneshvari Hospital, Shahid Beheshti, University of Medical Sciences, Tehran, Iran.

54. Primary Immunodeficiency Clinical Unit and Laboratory, Department of Dermatology, Venereology and Dermatooncology, Semmelweis University, Budapest, Hungary.

55. Department of Pediatrics, University Hospitals Leuven; KU Leuven, Department of Microbiology, Immunology and Transplantation; Laboratory for Inborn Errors of Immunity, KU Leuven, Leuven, Belgium.

56. Department of Pediatrics, Columbia University Irving Medical Center, New York, NY, USA.

57. University Clinic for Children's Diseases, Department of Pediatric Immunology, Medical Faculty, University “ St.Cyril and Methodij” Skopje, North Macedonia.

58. Department of Biomedicine, Aarhus University, Aarhus, Denmark.

59. Tokyo Medical & Dental University Hospital, Tokyo, Japan.

60. A*STAR Infectious Disease Labs, Agency for Science, Technology and Research, Singapore; Lee Kong Chian School of Medicine, Nanyang Technology University, Singapore.

61. National Institute of Allergy and Infectious Diseases, National Institutes of Health, Bethesda, MD, USA.

62. Laboratory of Medical Genetics, IRCCS Bambino Gesù Children’s Hospital, Rome, Italy.

63. Department of Biomedicine and Prevention, Tor Vergata University of Rome, Rome, Italy.

64. Comparative Immunology Group, School of Biochemistry and Immunology, Trinity Biomedical Sciences Institute, Trinity College Dublin, Ireland.

65. Department of Pediatrics, Graduate School of Biomedical and Health Sciences, Hiroshima University, Hiroshima, Japan.

66. Tokyo Medical and Dental University, Tokyo, Japan.

67. Department of Molecular Biology and Genetics, Bilkent University, Bilkent - Ankara, Turkey.

68. Haitian Study Group for Kaposi's Sarcoma and Opportunistic Infections (GHESKIO), Port-au-Prince, Haiti.

69. Institute of Biomedical Research of IdiPAZ, University Hospital “La Paz”, Madrid, Spain.

70. Center for Discovery and Innovation, Hackensack Meridian Health, Nutley, NJ, USA.

71. Department of Biosciences, Biotechnology and Biopharmaceutics, University of Bari A. Moro, Bari, Italy.

72. IIBB-CSIC, IDIBAPS, Barcelona, Spain.

73. Faculdades Pequeno Príncipe, Instituto de Pesquisa Pelé Pequeno Príncipe, Curitiba, Brazil.

74. Neurometabolic Diseases Laboratory, Bellvitge Biomedical Research Institute (IDIBELL), L'Hospitalet de Llobregat, Barcelona, Spain; Catalan Institution of Research and Advanced Studies (ICREA), Barcelona, Spain; Center for Biomedical Research on Rare Diseases (CIBERER), ISCIII, Barcelona, Spain.

75. Human Evolutionary Genetics Unit, CNRS U2000, Institut Pasteur, Paris, France; Human Genomics and Evolution, Collège de France, Paris, France.

76. Al Jalila Children's Hospital, Dubai, UAE.

77. University Hospital St. Marina, Varna, Bulgaria.

78. Department of Immunology, University Hospital of Gran Canaria Dr. Negrín, Canarian Health System, Las Palmas de Gran Canaria; Department of Clinical Sciences, University Fernando Pessoa Canarias, Las Palmas de Gran Canaria, Spain.

79. Department of Paediatric Infectious Diseases and Virology, Imperial College London, London, UK; Centre for Paediatrics and Child Health, Faculty of Medicine, Imperial College London, London, UK.

80. Department of Immunology, Second Faculty of Medicine Charles University, V Uvalu, University Hospital in Motol, Prague, Czech Republic.

81. Adult Immunodeficiency Unit, Infectious Diseases, Inflammation Center, University of Helsinki and Helsinki University Hospital, Helsinki, Finland; Rare Diseases Center and Pediatric Research Center, Children's Hospital, University of Helsinki and Helsinki University Hospital, Helsinki, Finland.

82. Specialized Immunology Laboratory of Dr. Shahrooei, Ahvaz, Iran; Department of Microbiology and Immunology, Clinical and Diagnostic Immunology, KU Leuven, Leuven, Belgium.

83. Department of Immunology, Dmitry Rogachev National Medical Research Center of Pediatric Hematology, Oncology and Immunology, Moscow, Russia.

84. Central European Institute of Technology & Department of Biology, Faculty of Medicine, Masaryk University, Brno, Czech Republic.

85. Department of Pharmacology & Molecular Therapeutics, Uniformed Services University of the Health Sciences, Bethesda, MD, USA.

86. Pediatric Infectious Diseases and Immunodeficiencies Unit, Vall d’Hebron Barcelona Hospital Campus, Barcelona, Catalonia, Spain.

87. St. Giles Laboratory of Human Genetics of Infectious Diseases, Rockefeller Branch, The Rockefeller University, New York, NY, USA; Department of Medical Microbiology, University Medical Center Utrecht, Utrecht, Netherlands.

88. Department of Internal Medicine II, Medical University of Innsbruck, Innsbruck, Austria.

89. Garvan Institute of Medical Research, Darlinghurst, NSW, Australia; St Vincent’s Clinical School, Faculty of Medicine, UNSW Sydney, NSW, Australia.

90. Bursa Uludag University, Faculty of Medicine, Department of Medical Genetics, Department of Histology & Embryology; Bursa Uludag University, Health Sciences Institute, Department of Translational Medicine

91. BC Children's Hospital, The University of British Columbia, Vancouver, Canada.

92. Centre for Precision Therapeutics, Genetics & Genomic Medicine Centre, NeuroGen Children's Healthcare and Lecturer, Holy Family Red Crescent Medical College Dhaka, Bangladesh.

93. College of Medicine, Mohammed Bin Rashid University of Medicine and Health Sciences, Dubai, UAE; Cellular Intelligence (Ci) Lab, GenomeArc Inc., Toronto, ON, Canada.

94. Department of Neurology, Amsterdam Neuroscience, Amsterdam University Medical Center, University of Amsterdam, Amsterdam, The Netherlands.

95. Department of Medicine, Division of Infectious Diseases, McGill University Health Centre, Montréal, Québec, Canada; Infectious Disease Susceptibility Program, Research Institute, McGill University Health Centre, Montréal, Québec, Canada.

96. Department of Pediatric Pneumology, Immunology and Intensive Care, Charité Universitätsmedizin, Berlin University Hospital Center, Berlin, Germany; Labor Berlin GmbH, Department of Immunology, Berlin, Germany; Berlin Institutes of Health (BIH), Berlin-Brandenburg Center for Regenerative Therapies, Berlin, Germany.

97. Department of General Internal Medicine, Medical Intensive Care Unit, University Hospitals Leuven, Leuven, Belgium.

98. Biosciences Institute, University of São Paulo, São Paulo, Brazil.

99. Molecular Biophysics Division, Faculty of Physics, A. Mickiewicz University, Poznań, Poland.

100. The Rockefeller University & Howard Hughes Medical Institute, New York, NY, USA; Necker Hospital for Sick Children & INSERM, Paris, France.
